# Electroacupuncture-Regulated miR-34a-3p/PDCD6 Axis Promotes Post-Spinal Cord Injury Recovery in Both *In Vitro* and *In Vivo* Settings

**DOI:** 10.1155/2022/9329494

**Published:** 2022-09-12

**Authors:** Lili Ma, Lizhong Ma, Yu Yang, Ting Chen, Limin Wang, Qilong Deng

**Affiliations:** ^1^Department of Infectious Medicine, Taizhou Hospital of Zhejiang Province Affiliated to Wenzhou Medical University, Linhai, 317000 Zhejiang Province, China; ^2^Rehabilitation Medical Center, Taizhou Hospital of Zhejiang Province Affiliated to Wenzhou Medical University, Linhai, 317000 Zhejiang Province, China; ^3^Department of Orthopedic Surgery, Taizhou Hospital of Zhejiang Province Affiliated to Wenzhou Medical University, Linhai, 317000 Zhejiang Province, China; ^4^Department of Dermatology, Taizhou Hospital of Zhejiang Province Affiliated to Wenzhou Medical University, Linhai, 317000 Zhejiang Province, China; ^5^Department of Internal Neurology, Taizhou Hospital of Zhejiang Province Affiliated to Wenzhou Medical University, Linhai, 317000 Zhejiang Province, China; ^6^Luqiao Hospital, Taizhou Enze Medical Center (Group), Taizhou, 318000 Zhejiang Province, China

## Abstract

Electroacupuncture (EA) could enhance neuroregeneration and posttraumatic conditions; however, the underlying regulatory mechanisms remain ambiguous. PDCD6 (programmed cell death 6) is an established proapoptotic regulator which is responsible for motoneuronal death. However, its potential regulatory role in post-spinal cord injury (SCI) regeneration has remained largely unknown. Further investigations are warranted to clarify the involvement of PDCD6 post-SCI recovery and the underlying mechanisms. In our study, based on bioinformatics prediction, we found that miR-34a-3p might be an upstream regulator miRNA for PDCD6, which was subsequently validated through combined utilization of the qRT-PCR, western blot, and dual-luciferase reporter system. Our *in vitro* results showed that miR-34a-3p might promote the *in vitro* differentiation of neural stem cell (NSC) through suppressing PDCD6 and regulating other important neural markers such as fibroblast growth factor receptor 1 (FGFR1), MAP1/2 (MAP kinase kinases 1/2), myelin basic protein (MBP), *β*III-tubulin Class III *β*-tubulin (*β*III tubulin), and glial fibrillary acidic protein (GFAP). Notably, in the post-SCI rat model, exogenous miR-34a-3p agomir obviously inhibited the expression of PDCD6 at the protein level and promoted neuronal proliferation, motoneurons regeneration, and axonal myelination. The restorations at cellular level might contribute to the improved hindlimbs functions of post-SCI rats, which was manifested by the Basso-Beattie-Bresnahan (BBB) locomotor test. The impact of miR-34a-3p was further promoted by EA treatment *in vivo*. Conclusively, this paper argues that a miR-34a-3p/PDCD6 axis might be a candidate therapeutic target for treating SCI and that the therapeutic effect of EA is driven through this pathway.

## 1. Introduction

Spinal cord injury (SCI) is caused by traumatic physical damage or chronic degenerative diseases depending on the etiology [1]. SCI is pathophysiologically manifested by excessive inflammation [2] and neuronal apoptosis [3]. Clinical consequences of SCI include either temporary or permanent loss of normal spinal functions [1] and, in severe cases, complete dependency and shortened life span [4], which severely affect the wellbeing of the sufferers in physical and emotional aspects and bring about heavy social and economic burden [4].

Over years, endeavors have been made to develop novel strategies for promoting functional rehabilitation following SCI, among which transplantation neural stem cells (NSCs) have emerged as a promising approach in the field of regenerative therapy [5]. NSCs are pluripotent cells with the potential to differentiate into various mature cells constituting the mammalian central nervous system (such as neurons, astrocytes, and oligodendrocytes) [6]. Due to their important neurological roles, NSCs are widely distributed throughout the spinal cord and respond differently under homeostatic condition or external stimulations [7]. An animal study showed that transplantation of NSCs stemmed from adult spinal cord facilitated the post-SCI recovery in rodents [7]. Acupuncture, a traditional Chinese medicine, has found a broad application in the treatment of various neurological diseases. As an improved variant of acupuncture approach, electroacupuncture (EA) had been reported to be efficacious in treating various SCI-related symptoms [8] [9–13]. Combined treatment of EA and curcumin improves the recovery of motor function and regulates oxidative stress in rats after traumatic SCI [14]. EA regulation of ApoE and Nrf2 to inhibit inflammation and oxidative stress in SCI has been also reported [15]. EA stimulation was reported to regulate the function of SCs, which favors axonal recovery post-SCI [16]. Through regulating Notch signaling pathway, EA was found to promote the recovery following SCI in rats [9]. The effectiveness of EA in treating trauma has also been demonstrated [17], but its possible mechanism in SCI still needs to be revealed. Moreover, whether EA would potentiate NSCs-induced post-SCI regeneration and the underlying mechanism needs to be explored.

PDCD6 (programmed cell death 6), or apoptosis-linked gene-2 (ALG-2), is an established regulator of apoptosis [18]. Mechanistically, PDCD6 works in concert with human death-associated protein kinase 1 (DAPK1) to trigger caspase-3 dependent signaling [19]. However, in the context of neuronal death, another interactor of PDCD6, namely ALG-2 interacting protein 1 (AIP1; also designated programmed cell death 6-interacting protein, Alix), has drawn more attention. First, AIP1 (ALG-2-interacting protein 1) itself was recognized as an important regulator of neuronal cell death [20]. Second, previous reports showed that PDCD6 could mediate motoneuronal death by forming a complex with AIP1 [21]. Furthermore, the binding between PDCD6 and AIP1 was crucial in the activation of both caspase-dependent and caspase-independent pathways that determined the survival of cerebellar granule neurons [22]. Given the foregoing, although the proapoptotic property of PDCD6 has been extensively studied in various contexts, its potential role in SCI has remained elusive. We speculated that PDCD6 might be responsible for SCI-associated neuronal death and aimed at clarifying this speculation.

miRNAs, endogenous noncoding RNAs composed of approximately 20 nucleotides [23], exert their multifunctional regulatory roles mainly through acting as epigenetic modulators [24]. By binding to their targeted mRNAs' 3′UTR (3′ untranslated region), miRNAs profoundly affect the posttranscriptional gene degradation or silencing [25]. In such a manner, the expression of the majority of protein-coding genes and the corresponding biological processes are under the control of miRNAs [26]. In previous reports, miRNAs were proposed to be essential for repairing SCI [26]; for example, bone marrow mesenchymal stem cells (BMSCs) displayed potential for repairing damaged spinal cord tissue in an *in vivo* rat model whereas reduced miR-127-5p expression was found to exacerbate SCI *via* activation of MAPK1 (mitogen-activated protein kinase 1) [27]. Aside from these regulatory roles in SCI, the importance of miRNA in SCI-induced apoptosis is frequently reported; miR-411 could alleviate post-SCI apoptosis and damage induced by inflammation [28]. Similarly, overexpression of miR-129-5p was found to alleviate SCI-related inflammation [29]. Specifically, in SCI rat model, miR-34a-3p was proposed to suppress the expression of CD47, thereby triggering PI3K/AKT signaling and ameliorate SCI-associated apoptosis [30]. In a more recent *in vivo* study, a reduced miR-34a-3p level was found following induction of SCI. By contrast, upregulating miR-34a-3p validly reduced inflammatory microglia count, accompanied by improved neural structures [31].

Collectively, both miR-34a-3p and PDCD6 were reported to be implicated in the pathophysiology of SCI through modulating apoptosis, and as far as we are aware, there is currently no evidence regarding the joint effect of miR-34a-3p and PDCD6 on post-SCI regeneration. Moreover, EA was found to enhance postischemic stroke neurobehavioral function *via* targeting of SOX2-mediated axonal regeneration by microRNA-132 [32]. Also, EA pretreatment is protective against ischemia/reperfusion injury *via* miR-214 [33] and miR-124 [34]. However, the underlying mechanism of EA and miR-34a-3p/PDCD6 axis in post-spinal cord injury recovery has never been studied. Hence, the motivation and novelty of the study is to focus on elucidating the potential miR-34a-3p/PDCD6 regulatory axis in NSCs, in order to provide novel perspective for SCI treatment and research. We also aimed to explore the regulatory effect of EA on this pathway *in vivo*.

## 2. Materials and Methods

### 2.1. Target Prediction Using Bioinformatics Tools

To provide a basis for subsequent investigations, we retrieved the 3′UTR sequence of PDCD6 from the NCBI Gene database. Moreover, the sequence of miR-34a-3p was retrieved from Targetscan (URL: http://www.targetscan.org/). The prediction of binding sites between miR-34a-3p and PDCD6 was carried out by online LncTar noncoding RNA target site prediction tools (URL: http://www.cuilab.cn/lnctar) using the default parameters.

### 2.2. Separation and Cultivation of NSCs as Neurospheres (NSs)

NSCs were extracted from diencephalon and telencephalon from rat at embryonic day 14.5 obtained from Shanghai SLAC Laboratory Animal Co., Ltd. (Shanghai, China) by dissecting microscope, mechanical pulverisation, and filtering with a 70 mm mesh filter. The extracted NSCs were seeded in a serum-free medium comprising DMEM/F12 (Invitrogen, Grand Island, NY), 100 IU/mL penicillin–streptomycin (Invitrogen, Grand Island, NY), 1% N2 supplement (Invitrogen), 20 ng/mL bFGF (Sigma-Aldrich), and 20 ng/mL EGF (PeproTech, Rocky Hill, NJ). The cells were seeded as detached NSs in low attachment six-well plates, with 4 mL medium/well; 2-3 mL of the medium was substituted each day. NSCs were subcultured at a frequency of 3–5 days *via* Accutase (Millipore, Bedford, MA) digestion in the medium. The cultures were performed in a 5% CO2 incubator at 37°C.

### 2.3. Immunocytochemistry Analysis to Characterize NSs

Immunocytochemistry was performed to characterize NSs *in vitro* with anti-Nestin antibodies (Abs) (a marker for NSCs, 19483-1-AP, ProteinTech), glial fibrillary acidic protein (GFAP) (a marker for astrocytes, 60190-1-Ig, ProteinTech), *β*III-tubulin (a marker for immature neurons, 11224-1-AP, ProteinTech), and Olig2 (a marker for immature oligodendrocytes, MABN50, MERCK). The nuclei in the NSs were treated with 4′-6-diamidino-2- phenylindole (DAPI) (Sigma-Aldrich, USA) counterstaining. PE- and FITC-conjugated secondary Abs (Jackson Laboratories, West Grove, PA, USA) were added, followed by imaging of NSs with confocal microscopy (Leica sp8, Germany).

### 2.4. Dual-Luciferase Reporter Assay

The *in silico* prediction of the binding site between miR-34a-3p and the 3′UTR of PDCD6 was retrieved from bioinformatics website TargetScan (URL: http://www.targetscan.org). Wild-type (WT) PDCD6 3′UTR fragment containing the putative miR-34a-3p binding sequence, along with the mutated version (MUT), was ordered from Sangon Biotech (Shanghai, China). The above sequences were cloned into the downstream of pLenti-UTR-Dual-Luciferase Cloning Vector (Applied Biological Materials, BC, Canada) to generate PDCD6-WT and PDCD6-MUT recombinant vectors. NSCs were plated in 6-well plates for cotransfection with PDCD6-WT/PDCD6-MUT plasmids and miR-34a-3p mimic. After 48 h of transfection, luciferase activity was quantified using a Spectramax® L Microplate Luminometer (Molecular Devices, CA, USA). Relative luciferase activity was calculated by normalizing the intensity of Renilla luciferase against the internal control firefly luciferase.

### 2.5. *In Vitro* miRNA Mimic or Inhibitor Transfection

miR-34a-3p mimics or inhibitor, synthesized by Sangon Biotech (Shanghai, China), was used to induce transient *in vitro* or *in vivo* miR-34a-3p overexpression or knockdown. For *in vitro* miRNA transfection, the suspended NSCs (passage < 3) were seeded onto the Costar® 24-well plate (Corning, NY, USA) at a density of 10,000 cells per well and maintained in complete culture medium for 2 d until the NSCs monolayer reached a confluency of 50%. Then, Lipofectamine 2000™ reagent (Thermo Fisher Scientific, USA) was used as a vector for miRNA transfection. Next, NSCs in different wells underwent transfection with miR-34a-3p mimics or inhibitor or the corresponding negative control (NC) (sequence-scrambled oligonucleotides). The cell specimens were harvested at 48 h posttransfection for various downstream analyses unless specified otherwise.

### 2.6. Western Blotting (WB) Analysis

WB analysis was employed to quantify the protein expression of various neural markers in NSCs and spinal tissues from SCI model. Briefly, each sample was first lysed by RIPA buffer (Beyotime, Shanghai, China) and the resulting lysate was harvested through centrifugation at 4°C, 12000g for 5 min. After boiled water bath with sample loading buffer, the denatured total proteins were subjected to sodium dodecyl sulfate-polyacrylamide gel electrophoresis (SDS-PAGE) isolation according to their differences in molecular weight. Next, the proteins were electrically transferred onto polyvinylidene difluoride (PVDF, Millipore, CA, USA) membranes. After blocking with skimmed milk for 2 h in room temperature, the PVDF membranes underwent incubation with primary Abs at 4°C overnight, which was followed by incubation with secondary Abs (1 : 1000) under the same condition. Protein bands were visualized *via* SuperSignal™ WB Enhancer (Thermo Fisher Scientific, USA). The intensity of each protein band was quantified using the ImageJ software (NIH ImageJ; NIH, Bethesda, MD). The expression of proteins of interest was normalized with *β*-actin. The level of protein phosphorylation was calculated by normalizing against the corresponding total protein. Aside from anti-MEK1/2 (9122S) and anti-phospho-MEK1/2 (9121S) that were supplied by cell signaling technology (Danvers, MA, USA) and NF-200 (N4142) purchased from Millipore (Temecula, CA, USA), the other Abs used in this study were obtained from Abcam (Cambridge, UK), including anti-PDCD6 (ab109181), anti-*β*III-tubulin (ab18207), anti-GFAP (ab7260), anti-MBP (ab209328), and anti-FGFR1 (ab76464).

### 2.7. Quantitative Real-Time Polymerase Chain Reaction (qRT-PCR)

qRT-PCR was employed to evaluate miR-34a-3p expression in differentiating NSCs and in the specimens isolated from the spinal cord of post-SCI model. Total RNA extraction was carried out with TRIzol reagent (Invitrogen, CA, USA) in compliance with the supplier's instruction. Next, the purified RNA was treated with reverse transcription using the PrimeScript RT reagent kit (TaKaRa, Tokyo, Japan). The generated cDNA was employed in qRT-PCR which was executed using the SYBR Green qPCR Master Mix (QIAGEN, Hilden, Germany). The primer pairs specific for miR-34a-3p/U6 (internal control for miRNA) were ordered from Sangon Biotech (Shanghai, China). 2^−*ΔΔ*Ct^ [35] calculated the miR-34a-3p expression (normalized against U6). The primer pairs used in qRT-PCR were as follows:

miR-34a-3p: F: 5′-GGGATCCCGTCATATGAAC-3′; R: 5′-GTGCAGGGTCCGAGGT-3′; U6: F: 5′-TTGGTGCTCGCTTCGGCA-3′; R: 5′-GTGCAGGGTCCGAGGT-3′.

### 2.8. Immunofluorescence Assay

Immunocytochemistry was used to visualize the intracellular localization of the proteins of interest. NSCs were subjected to fixation with 4% paraformaldehyde (PFA, TCI technology, Shanghai, China) and subsequent permeabilization in 0.3% Triton™ X-100 (Thermo Scientific, CA, USA). For frozen spinal cord tissues pretreatment, samples were first homogenized in room temperature for approximately 15 min and were subsequently soaked in precooled PBS for about 10 min; the antigenicity was then restored by incubation with proteinase K (Thermo Fisher, USA). After pretreatment, 5% Gibco™ bovine serum albumin (Gibco, USA) was used to bind to nonspecific binding sites in both cell and tissue samples. The incubation of primary Abs was carried out overnight at 4°C. After PBS rinsing, the signal of the primary Abs was amplified by Alexa Fluor 488 (for GFAP)/568 (for NF200)-conjugated secondary Abs (1 : 500; Life Technologies, USA), with the simultaneous counterstaining of nucleus using DAPI. An Olympus BX63 fluorescence microscope (Olympus, Tokyo, Japan) was utilized to observe the localization of proteins of interest.

### 2.9. EdU Proliferation Measurement

NSCs were divided into 1 d and 3 d groups according to the time from miRNA mimic or inhibitor transfection. After reaching confluency, the monolayer NSCs were detached, resuspended, and seeded onto a 96-well plate (Sigma-Aldrich, MO, USA) at a density of 10,000 per well. 20 *μ*M 5-ethynyl-2′-deoxyuridine (EdU, Thermo Fisher Scientific) was placed into the plate. Following a 1 d incubation period, NSCs were fixed by precooled phosphate-buffered saline (PBS) containing 4% paraformaldehyde (PFA, HEAD biotechnology, Beijing, China). Subsequently, the fixed NSCs underwent incubation with Apollo® reaction cocktails for 30 min under room temperature (22 ± 2°C), followed by DAPI counterstaining. NSCs were then captured in randomly selected field under the Olympus BX63 fluorescence microscope. The NSC proliferation was described as ratio of EdU-stained cells against DAPI-positive cells (total number of cells).

### 2.10. Establishment of Rat SCI Model

Female [36, 37] SD rats (weighing 200 ± 20 g, aged 7 ± 1 weeks) were ordered from Shanghai SLAC Laboratory Animal Co., Ltd. (Shanghai, China). SD rats were maintained in standard cages under a specific pathogen-free (SPF) environment, with free access to food and water ad libitum for approximately one week to acclimatize. To establish a rat SCI model for investigating the NSC-induced post-SCI recovery, the modified Allen's method (a weight-drop procedure) was employed [38]. For SCI treatment, SD rats (*n* = 60) were anesthetized with isoflurane (1%oxygen + 5%isoflurane) and were shaved at the T10 vertebral level where the surgery was performed. The surgery began with a longitudinal incision made by a sterilized scalpel, the muscular plexus of the back was subsequently dissected with surgical instruments, and the lamina and spinous process was removed to expose the T10 spinal dura. Next, the SCI (contusion) centered on the T10 vertebral level was induced by a 10 g steel rod 5 cm above the target site; this process was controlled by a W.M.Keck Center Impactor Model III (Rutgers, NJ, USA). The sham operation group was used as control and received only laminectomy (*n* = 6) under isoflurane (1%oxygen + 5%isoflurane). After confirming hemostasis, the muscle/skin incisions were successively sutured in layers sequentially. The successful establishment of SCI model was determined through the following criteria: (i) observable hyperemia around the lesion site; (ii) whole-body tremor; (iii) spasmodic tail flick; and (iv) sluggish movement or paralysis of the hindlimbs. Postoperatively, antibiotics were injected intramuscularly for 3 days. All the rats were successfully modeled. Next, 60 post-SCI rats were randomized into 10 subgroups (*n* = 6 each): (1) SCI, (2) SCI+EA, (3) SCI+agomir NC, (4) SCI+miR-34a-3p agomir, (5) SCI+EA+agomir NC, (6) SCI+EA+miR-34a-3p agomir, (7) SCI-3D, (8) SCI-7D, (9) NC-agomir 7D, and (10) miR-34a-3p agomir 7D. For SD rats receiving EA or agomir treatment (SCI, SCI+EA, SCI+agomir NC, SCI+miR-34a-3p agomir, SCI+EA+agomir NC, and SCI+EA+miR-34a-3p agomir, a total number of 36 rats), tissues were harvested at the end point of EA treatment or BBB score observation. For SD rats used to observe time-course dependent alteration of PDCD6 and miR-34a-3p (SCI-3D, SCI-7D, NC-agomir 7D, and miR-34a-3p agomir 7D, a total number of 24 rats), tissues were harvested 3 d/7 d after the induction of SCI.

### 2.11. Acupuncture Treatment

Electroacupuncture (EA) is a modern version of a traditional Chinese medical therapy, which has been widely utilized for various research purposes [39] [40, 41]. The animals achieved physical stabilization 3 d after the SCI operation and were subjected to an EA stimulation which was performed on GV4 (“Mingmen,” posterior midline underneath the spinous process of the 2nd lumbar vertebra) and GV14 (“Dazhui,” posterior midline underneath the spinous process of the 7th cervical vertebra). Briefly, the rats were firmly fixed on wooden boards. Two acupuncture needles (HANS-200E, Jisheng Medical Instruments, China) (0.3 × 25 mm) were inserted to a depth of 5~7 mm at each acupoint, whereby in a continuous-wave of 2 Hz frequency, 0.4 mA intensity was produced. The EA were applied on a daily basis, 5 days per week for a total of 4 consecutive weeks, each time lasting for 20 min. All the above procedures were conducted after isoflurane (1%oxygen + 5%isoflurane).

### 2.12. *In Vivo* miRNA Transplantation

miR-34a-3p-agomir (and the corresponding NC-agomir), which was specifically designed as an enhanced version of miR-34a-3p agomir to ensure the biostability and mRNA-inhibitory efficacy in an *in vivo* setting, was used to investigate the *in vivo* biological impact of miR-34a-3p on post-SCI recovery. Briefly, miR-34a-3p-agomir and the corresponding NC were treated with saline solution dilution to reach a final concentration of 15 nmol/mL. The miR-34a-3p or NC-agomir solutions were filled into the abovementioned minipumps, allowing for the continuous delivery of the solution (at a rate of 1 *μ*L per hour) into the spinal cord through the mini-pump connected catheter [42]. The treatment was performed on a daily basis for three consecutive days. After the implantation, the incisions were sutured immediately, and rats were allowed to recover from the operation under an appropriate environment.

### 2.13. Basso-Beattie-Bresnahan (BBB) Score Assessment

The BBB locomotor test is an evaluation standard designed specifically for lumbar injury animal models, which assigns a score (proportional to the degree of recovery of hindlimb function) to each subject. The BBB test was carried out to estimate the post-SCI recovery of hindlimb functions at 1, 3, 7, 14, and 28 days post SCI. SD rats from each group were observed individually in a quiet, open-field setting that permitted free movement. The locomotor functions at various sites including the hip joints, ankle, and knee were examined by blinded trained observers according to the open-field BBB locomotion test [43] for 5 min to evaluate each rat's BBB score. The records for each group of animals were subjected to further statistical analyses. The control group was the sham-operated animals without functional impairment (BBB score: 21).

### 2.14. Fluoro-Gold Retrograde Tracing

For Fluoro-Gold (FG) retrograde tracing, the SD rats first underwent anesthesia, before a dorsal laminectomy was carried out between the T11 and 12 vertebral level. Approximately 3 *μ*L of Fluoro-Gold™ (FG; Biotium, CA, USA) was slowly injected into the site with newly induced SCI (at approximately 3 mm of spinal cord caudal to the initial SCI lesion). On the second day post operation, the FG-labeled SD rats were perfused, allowing for separation of a spinal segment (10 mm in length) containing the transection site at T10 vertebral level. The separated specimens were longitudinally sectioned into slices *via* a freezing microtome (Thomas Scientific, NJ, USA). Then, the FG-labeled cells were inspected and counted at five different sites.

### 2.15. Toluidine Blue Staining Analysis

The spinal cord tissues embedded in paraffin were sectioned into 4 *μ*m slices and then stained with toluidine blue staining. The tissue sections were dewaxed into distilled water. Toluidine blue working solution (5 mL 1%toluidine blue stock solution + 45 mL 1%sodium chloride solution) was used for dying for 2 min, followed by washing with distilled water for three times. Next, treatment with 0.5% acetic acid for 1 min was performed, followed by rinsing, dehydration, clearing, and sealing.

### 2.16. Transmission Electron Microscopy (TEM)

To evaluate myelination, rats (*n* = 6 per group) were anesthetized for 2.5% glutaraldehyde (Kemiou, Tianjing, China; 25%glutaraldehyde : 4%PFA = 1 : 9) perfusion eight weeks postoperatively. The collected tissue samples were then sequentially subjected to 2.5% glutaraldehyde fixing, 1% osmium tetroxide treatment, and cleaning in a series of propylene oxide post dehydration. Subsequently, 50 nm ultrathin sections were double-stained with 2% led citrate and uranylacetate for TEM (HITACHI, HT7800) observation. The morphology of myelin was observed under TEM in a blinded manner.

### 2.17. Statistics and Analysis

All statistical analyses were performed by GraphPad Prism8 (CA, USA), and the data were presented as mean ± standard deviation (SD). The one-way ANOVA approach was used to measure the statistical significance of one variable among three or more groups, followed by Tukey's post hoc multiple comparison test, while the two-way ANOVA was applied for two or multiple variables in multiple-groups comparisons, followed by Šídák's multiple comparisons test. As to the BBB score, two-way ANOVA (repeated ANOVA) followed by the Tukey's multiple comparison test was carried out. Analysis results with *p* < 0.05 were assumed to be statistically significant.

## 3. Results

The results of the statistical tests performed in this study are reported in Additional File 1. The results of the experiments are presented below.

### 3.1. Characterization of NSCs

**Figure 1:**
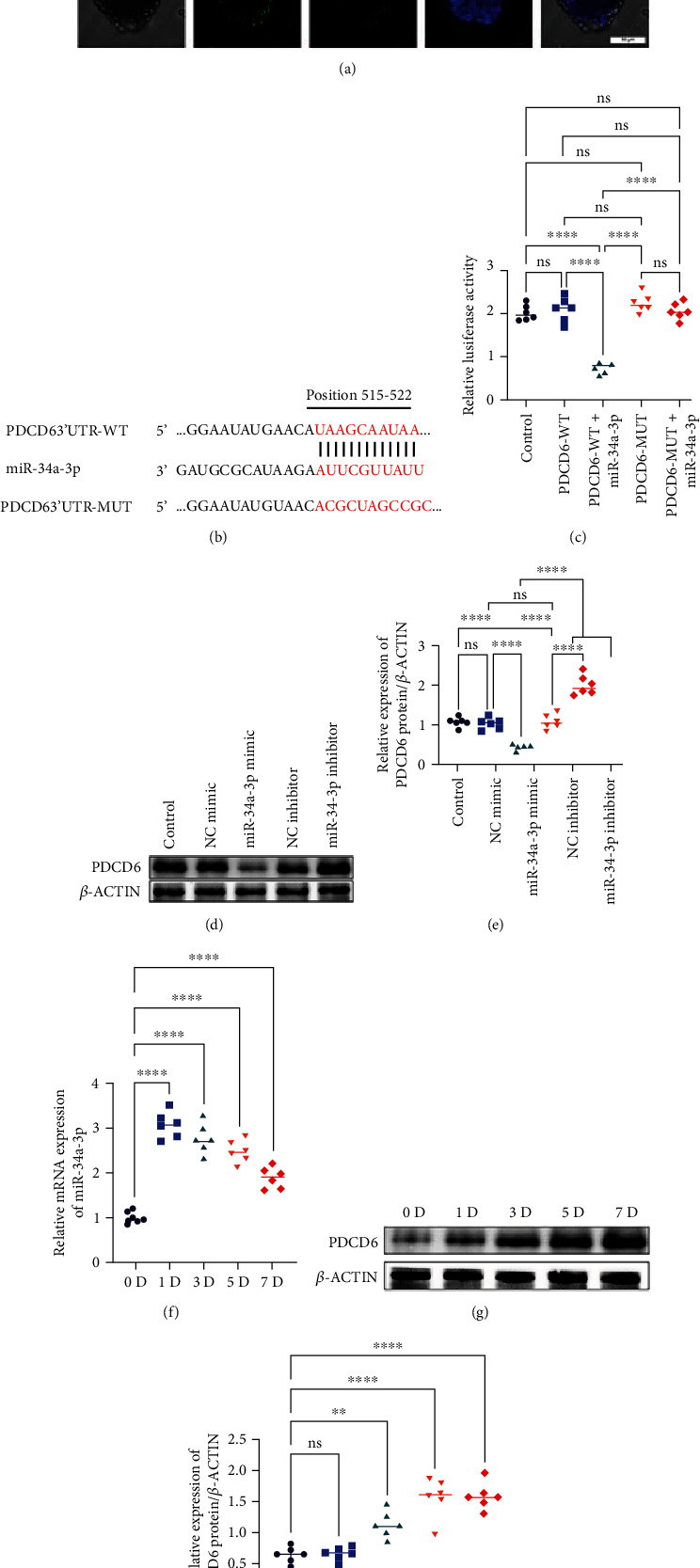
miR-34a-3p suppressed PDCD6 expression in differentiating NSCs time-dependently. (a) Phenotypic characterization of isolated NSCs. The cells were stained for Nestin, GFAP, *β*III tubulin, and Olig2 to determine their expression on NSCs. The representative images of six (*n* = 6) independent experiments are shown for each group. (b) miR-34a-3p directly binds to the 3′UTR of PDCD6 (position 737-743); the sequence marked in red represents the predicted miR-34a-3p binding site (with vertical lines indicating base pairing underneath) or mutated miR-34a-3p binding site (unable to bind to the miR-34a-3p sequence). (c) Relative luciferase activity of Renilla luciferase versus firefly luciferase in different groups. The experiments were performed in triplicate and repeated six times (*n* = 6 independent experiments). (d) Western blot analysis of PDCD6 protein expression in different treatment groups. Representative images of three independent experiments are presented. (e) Quantification of western blot bands of PDCD6. The gray values of six (*n* = 6) independent experiments were plotted. (f) qRT-PCR evaluation of NSCs differentiation-dependent evolution of miR-34a-3p expression at different time points. The experiment was done in triplicate and repeated six times. (g) Western blot analysis of PDCD6 protein expression in differentiating NSCs. Representative images of six (*n* = 6) independent experiments were presented. (h) Quantification results of western blot analysis of PDCD6. The experiments were performed in triplicate and repeated six times (*n* = 6 independent experiments). For the luciferase, qRT-PCR, and western blotting experiments, the one-way ANOVA followed by the Tukey's multiple comparisons test was utilized to detect the differences among the control group or 0 d group and the other groups. ^∗∗^*p* < 0.01, ^∗∗∗^*p* < 0.001, ^∗∗∗∗^*p* < 0.0001, and ns: nonsignificant among the compared groups.

NSC characterization was performed using immunocytochemical staining for Nestin, GFAP, *β*III tubulin, and Olig2 (Figure 1(a)). The cells were grown in NSs, and the immunohistochemical staining indicated that cells were strongly Nestin- and GFAP-positive, but low expression levels of *β*III-tubulin and Olig2 were recorded (Figure 1(a)), confirming that the isolated cells were NSCs.

### 3.2. miR-34a-3p Might Regulate *In Vitro* Differentiation of NSC *via* Suppressing PDCD6

The majority of miRNAs exert regulatory roles *via* binding to their target sites in 3′UTR of mRNAs [44]. We first predicted the direct interaction between miR-34a-3p and PDCD6 and found a consensus sequence as a binding site of miR-34a-3p in the 3′UTR of PDCD6 (Figure 1(b)). To further verify whether miR-34a-3p regulates PDCD6, we cloned the wild-type (WT) or mutant (MUT) PDCD6 3′UTR segments containing the predicted miR-34a-3p binding site (Figure 1(b)) into the dual-luciferase reporter vector downstream region. Luciferase activity assay indicated, as shown in Figure 1(c), that cotransfection of miR-34a-3p mimic and WT PDCD6 3′UTR significantly decreased the luciferase activity by comparison with the control and PDCD6-WT groups, and no difference was found between the MUT PDCD6 3′UTR+ miR-34a-3p mimic cotransfection and the control and PDCD6-WT groups, which confirmed a direct interaction between miR-34a-3p and PDCD6. This result was further corroborated by WB analysis at the protein level; and as shown in Figures 1(d) and 1(e), miR-34a-3p mimic obviously suppressed the expression of PDCD6.

We next examined whether miR-34a-3p could induce time-course dependent effects on NSC differentiation. To this end, proliferating NSCs (day 0) and differentiating NSCs (day 1, 3, 5, or 7) were collected from the culture flask for subsequent qRT-PCR and WB analyses. miR-34a-3p expression was increased in differentiating NSCs compared to the proliferating cells, with high expression levels in the early stages of differentiation (1 d~5 d) (Figure 1(f)). Moreover, the WB analysis revealed the gradual increase of PDCD6 protein expression during NSC differentiation, reaching a plateau at approximately 5 d (Figures 1(g)–1(h). The opposite trend between PDCD6 and miR-34a-3p expression signposted that miR-34a-3p might regulate NSC differentiation *via* targeting PDCD6.

### 3.3. miR-34a-3p Promotes NSC Proliferation and Neuronal Differentiation *In Vitro*

**Figure 2:**
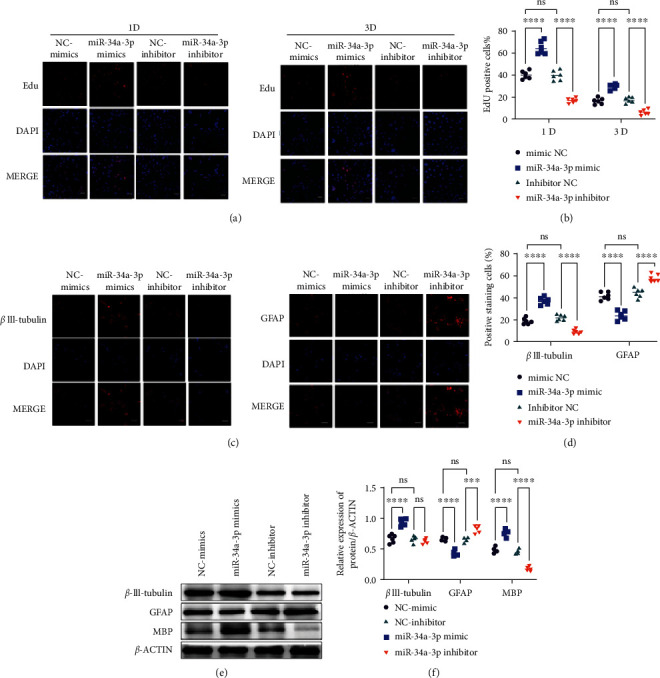
miR-34a-3p facilitates *in vitro* NSC proliferation/neuronal differentiation. (a) EdU cell proliferation assay of NSCs at different time points after treatment with miR-34a-3p mimic or inhibitor. Representative images of six (*n* = 6) independent experiments are presented. (b) Quantification of EdU-positive NSCs. (c) Immunofluorescence analysis of *β*III-tubulin and GFAP in different treatment groups. Representative images of six (*n* = 6) independent experiments were presented. (d) Quantitative representation of *β*III-tubulin- and GFAP-positive cells in different treatment groups as obtained from immunofluorescence analysis. (e, f) Western blot analyses and quantification of 3 important markers of neuronal differentiation (MBP, *β*III-tubulin and GFAP). Representative images of six (*n* = 6) independent experiments were presented. For the EdU, immunofluorescence, and western blotting experiments, two-way ANOVA followed by the Šídák's multiple comparisons test were utilized to identify differences. ^∗^*p* < 0.05, ^∗∗^*p* < 0.01, ^∗∗∗^*p* < 0.001, ^∗∗∗∗^*p* < 0.0001, and ns: nonsignificant among the compared groups. Scale bars = 20 *μ*m.

To investigate the proliferative impact of miR-34a-3p, we used the EdU assay to evaluate the proliferation of miR-34a-3p mimics or inhibitor transfected NSCs at 1 d and 3 d of differentiation (Figure 2(a)). At both time points, the ratio of EdU (red)-positive cells was calculated (Figure 2(b)). Compared to the NCs, the percentage of EdU-positive cells was increased by miR-34a-3p mimic while the opposite was observed with the miR-34a-3p inhibitor at both time points (Figures 2(a) and 2(b)). These results indicated the proproliferative effect of miR-34a-3p on NSCs.

Subsequently, we examined the potential role played by miR-34a-3p in regulating the self-renewal and differentiation ability of NSCs. Three important markers of the differentiation NSCs, including neuron-specific marker Class III *β*-tubulin (*β*III tubulin), astrocyte-specific marker GFAP, and oligodendrocyte specific marker myelin basic protein (MBP), were evaluated in miR-34a-3p mimic or inhibitor transfected NSCs at 5 d. The results of immunostaining against *β*III tubulin or GFAP are shown in Figure 2(c), along with the quantification results (Figure 2(d)); we found that in the miR-34a-3p mimic group, oligodendrocyte differentiation (manifested by the expression of *β*III tubulin) was significantly increased while astrocyte differentiation (manifested by the expression of GFAP) was significantly inhibited; the opposite results were found in the miR-34a-3p inhibitor group compared to the NC groups. Moreover, WB results (Figures 2(e) and 2(f)) indicated that *β*III tubulin and MBP were increased by miR-34a-3p overexpression but decreased by miR-34a-3p inhibitor; the contrary trends were observed for GFAP. These evidences jointly indicated that miR-34a-3p was involved in the differentiation of NSCs toward neural cells or oligodendrocytes but not toward astrocytes.

### 3.4. SCI-Associated Signaling Pathways Were Regulated by the miR-34a-3p/PDCD6 Axis

**Figure 3:**
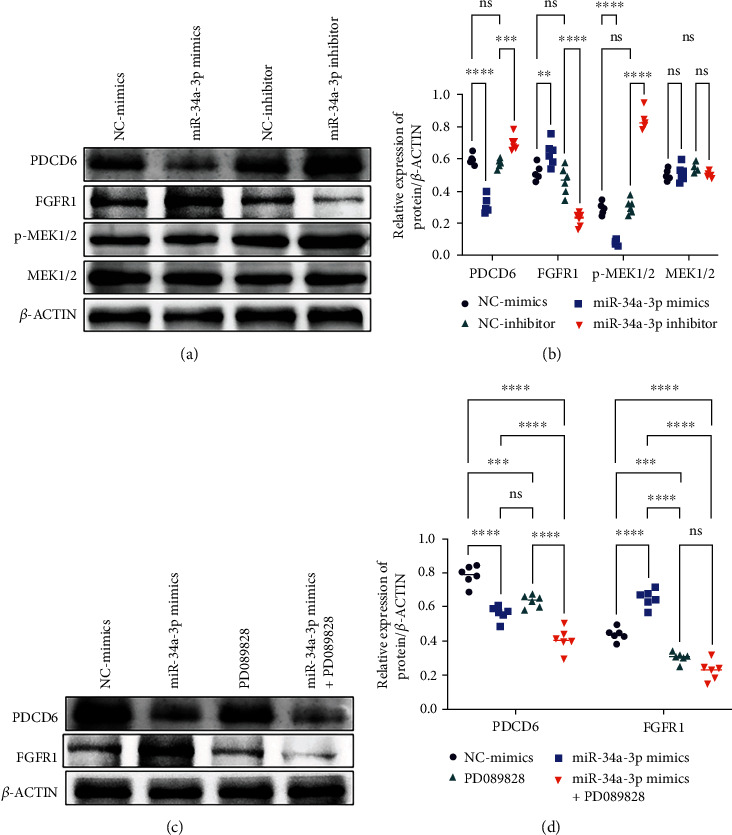
miR-34a-3p might regulate NSCs through FGFR1 or MEK1/2 signaling. (a, b) Western blot analyses and quantification showing the expression of FGFR1 and PDCD6 MEK1/2 and p-MEK1/2 after miR-34a mimic or inhibitor treatments. (c, d) Western blot analyses and quantification of PDCD6 and FGFR1 protein expression levels after treatment with miR-34a-3p mimic or PD089828 (a competitive inhibitor of FGFR1) or their combined treatment. The experiments were performed in triplicate and repeated six times (*n* = 6); representative images of three independent experiments were presented. Two-way ANOVA followed by the Šídák's multiple comparisons test was utilized to identify differences. ^∗^*p* < 0.05, ^∗∗^*p* < 0.01, ^∗∗∗^*p* < 0.001, ^∗∗∗∗^*p* < 0.0001, and ns: nonsignificant among the compared groups.

Since we have confirmed the inverse regulation of miR-34a-3p on PDCD6 expression, we further investigated the contribution of miR-34a-3p/PDCD6 cascade to SCI-associated canonical signaling pathways such as FGFR-signaling pathway which was shown to be counteract SCI-induced injury [45], as well as MEK1/2 whose hyperphosphorylation was considered a hallmark of SCI [46]. The results are shown in Figures 3(a) and 3(b), miR-34a-3p mimics induced a significant upregulation of FGFR1, and at the same time, inhibited the phosphorylation of MEK1/2 and downregulated PDCD6 expression; the miR-34a-3p inhibitor induced opposite effects. These evidences suggested that miR-34a-3p may promote the post-SCI recovery mainly through suppressing PDCD6 and promoting FGFR expression while inhibiting MEK1/2 phosphorylation. The subsequent treatment of miR-34a-3p mimic-transfected NSCs with PD089828 (a competitive inhibitor of FGFR1) demonstrated that PD089828 significantly reversed miR-34a-3p-induced increase of FGFR1 (Figures 3(c) and 3(d)).

### 3.5. EA Promotes miR-34a-3p/PDCD6 Axis-Facilitated Post-SCI Neural Regeneration in Rats

**Figure 4:**
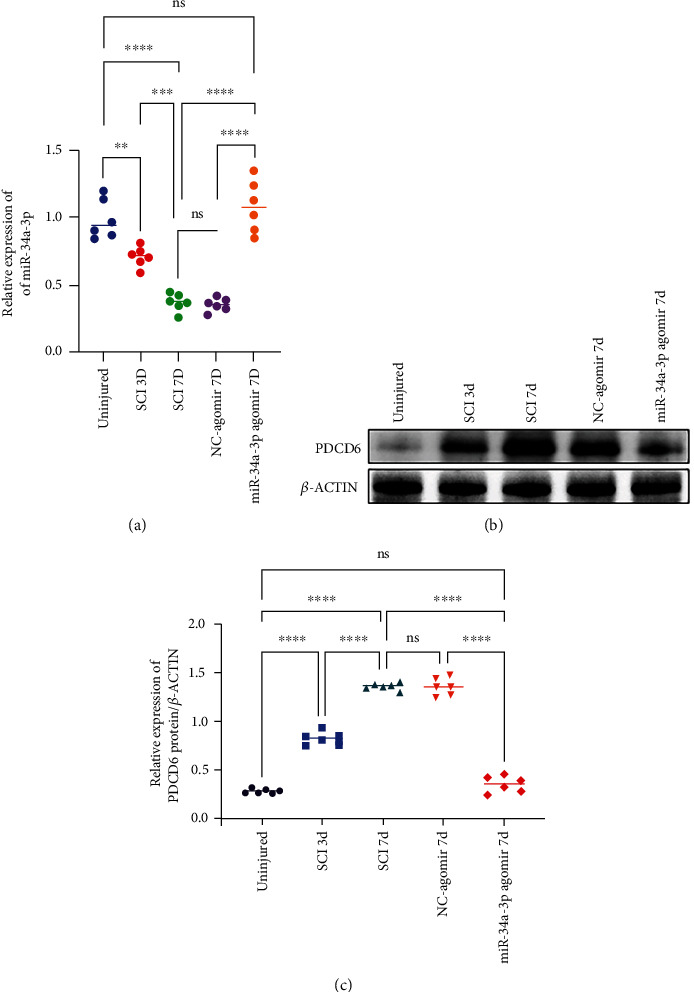
miR-34a-3p effectively facilitated post-SCI axonal regeneration and functional recovery *via* targeting PDCD6. (a) qRT-PCR quantification of miR-34a-3p expression at different post-SCI time points (3 d and 7 d); the comparison was also made against forced miR-34a-3p overexpression (as miR-34a-3p dropped markedly at 7 d post-SCI). The experiment was performed in triplicate from each of the six animals in each group. The average values obtained from each animal were used in statistical analysis and plotting. (b, c) Western blotting analysis and PDCD6 quantification in SCI rats. The western blotting analysis and quantification revealed that PDCD6 expression was elevated in a time-dependent fashion after the induction of SCI; the obvious miR-34a-3p overexpression at 7 d obviously reversed the SCI-induced PDCD6 elevation. Actin was used as an internal control. The experiments were performed in triplicate from 6 different animals (*n* = 6). One-way ANOVA followed by Tukey's multiple comparisons test was utilized to identify differences. ^∗∗^*p* < 0.01, ^∗∗∗^*p* < 0.001, ^∗∗∗∗^*p* < 0.0001, and ns: nonsignificant among the compared groups.

**Figure 5:**
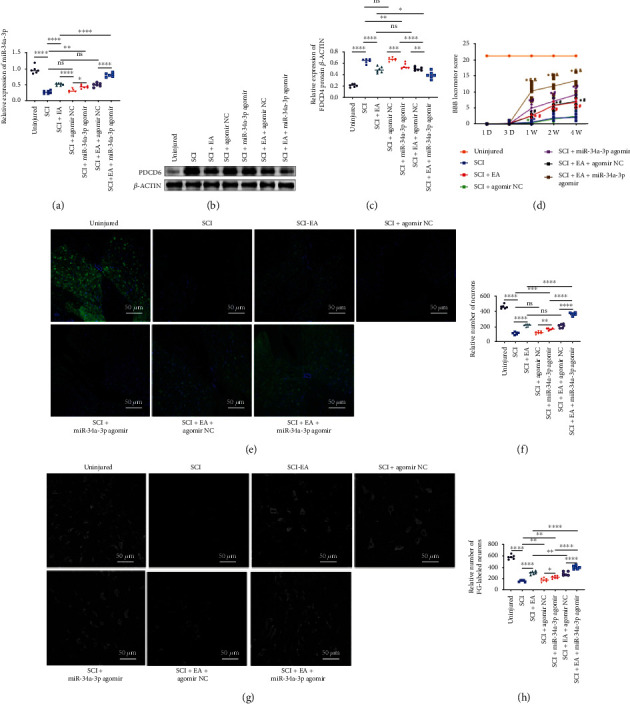
EA upregulates miR-34a-3p to exert post-SCI regenerative effects comparable to EA stimulation through suppressing PDCD6. (a) qRT-PCR analysis of PDCD6 expression after treatment with SCI with and without EA stimulation and miR-34a-3p agomir treatment conditions. (b) Western blotting analysis of PDCD6 expression. (c) Quantitative analysis of PDCD6 levels. (d) Determination of the relative BBB locomotor score in different treatment groups. (e) Analysis of the continuity of neurofilament. (f) Quantification of the number of neurons after staining for continuity analysis. (g) Analysis of regenerative neuron by FG labeling. (h) Quantification of FG-labeled neurons. Scale bars = 50 *μ*m. Except for (d), one-way ANOVA followed by the Tukey's multiple comparison test were utilized to identify differences. ^∗^*p* < 0.05, ^∗∗^*p* < 0.01, ^∗∗∗^*p* < 0.001, ^∗∗∗∗^*p* < 0.0001, and ns: nonsignificant among the compared groups. For (d), repeated measures two-way ANOVA followed by Tukey's multiple comparison test were utilized and ^∗^*p* < 0.05 versus the sham-operated (uninjured) group; ^#^*p* < 0.05 versus the SCI group; ^&^*p* < 0.05 versus the SCI+EA+agomir NC group. All the experiments were performed in triplicate from six animals (*n* = 6 animals/group).

Our *in vitro* study suggested that PDCD6 is a pivotal target for miR-34a-3p; hence, we further investigated these observations *in vivo*. As shown in Figure 4(a), we first found a time-course- (3 d and 7 d after SCI induction) dependent decrease of miR-34a-3p expression in post-SCI spinal cord tissue, which coincided with a gradually increased PDCD6 expression at protein level (Figures 4(b) and 4(c)). Given the notably lower miR-34a-3p expression in SCI-7D group compared with SCI-3D group, we selected this time point for miR-34a-3p agomir treatment. As expected, at the 7^th^ day after SCI induction, we found that SCI upregulation of PDCD6 was abolished by the treatment with miR-34a-3p agomir (Figures 4(b) and 4(c)), further confirming the *in vitro* results of the interaction between miR-34a-3p and PDCD6. In the subsequent combined treatment of SCI injury with EA and miR-34a-3p-agomir, we found that both EA and miR-34a-3p-agomir stimulated miR-34a-3p expression compared to the SCI group, and this effect was further promoted by the combined treatment of EA and miR-34a-3p-agomir (Figure 5(a)). Moreover, both EA and miR-34a-3p-agomir displayed inhibitory effects on PDCD6 expression at protein level (Figures 5(b) and 5(c)), and the effect of their combination treatment was greater than individual treatments. As reflected by BBB score (Figure 5(d)), the post-SCI hindlimb function was greatly restored after miR-34a-3p-agomir treatment. At the end of the 4-week-long observational period, NF-200 immunostaining showed the continuity of neurofilament in the SCI lesion center. Regenerating neuron fibers were rarely observed in SCI and SCI+NC-agomir groups (Figures 5(e) and 5(f)). In contrast, treatment with EA or miR-34a-3p agomir, either separately or in combination, increased the number of neuron fibers compared to the SCI and SCI+NC-agomir groups, and the combined effect of EA and miR-34a-3p agomir outperformed the treatment with EA or miR-34a-3p agomir single separated treatments (Figure 5(f)). By using FG retrograde tracing, we also examined the plasticity of axons extending from regenerated neurons. As shown in Figures 5(g) and 5(h), a relatively limited number of FG-labeled neurons were observed in both SCI and SCI+NC-agomir groups compared to that in EA- and miR-34a-3p agomir-treated groups. EA and miR-34a-3p exhibited a synergistic effect in promoting motor neuron regeneration (Figure 5(h)).

### 3.6. EA Promotes miR-34a-3p-Agomir-Facilitated Axonal Myelination

**Figure 6:**
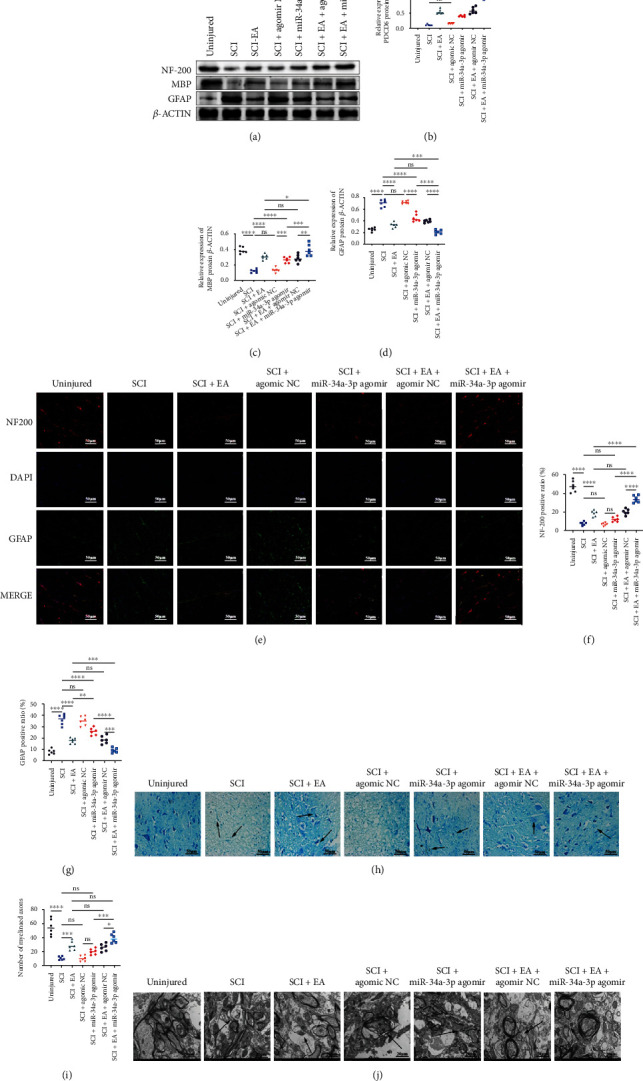
EA upregulates miR-34a-3p to promote post-SCI axonal myelination. (a–d) Western blotting analysis and quantification of GFAP, NF-200, and MBP protein levels under EA stimulation and miR-34a-3p agomir treatment following SCI. Six experiments were performed in triplicate from six animals (*n* = 6 animals/group)), and the representative images are shown. The gray value of bands from each band relative to *β*-actin is presented in the graphs. (e–g) Immunofluorescence assay of NF200 and GFAP and quantitative analysis of NF200- and GFAP-positive ratios under EA stimulation and miR-34a-3p agomir treatment following SCI. Representative images of stained tissues sections from six different animals per group are shown. The quantitative analysis showed the positive ratio of each protein; scale bars = 50 *μ*m. (h) Toluidine blue staining showing that the number of myelinated axons was significantly elevated in response to either EA stimulation or treatment with miR-34a-3p agomir following SCI, scale bars = 50 *μ*m. Representative images of stained tissues sections from six different animals per group (*n* = 6 animals/group) are shown. (i) Quantification of the myelinated axons; values from each of the six animals in each group are presented. (j) Viable myelinated axons in the spinal cord under the scanning electron microscope, scale bar = 20 *μ*m. Representative images of stained tissues sections from six different animals per group are shown. One-way ANOVA followed by the Tukey's multiple comparisons test was utilized to identify differences. The arrows indicated the injury parts; ^∗^*p* < 0.05, ^∗∗^*p* < 0.01, ^∗∗∗^*p* < 0.001, ^∗∗∗∗^*p* < 0.0001, and ns: nonsignificant among the compared groups.

**Figure 7:**
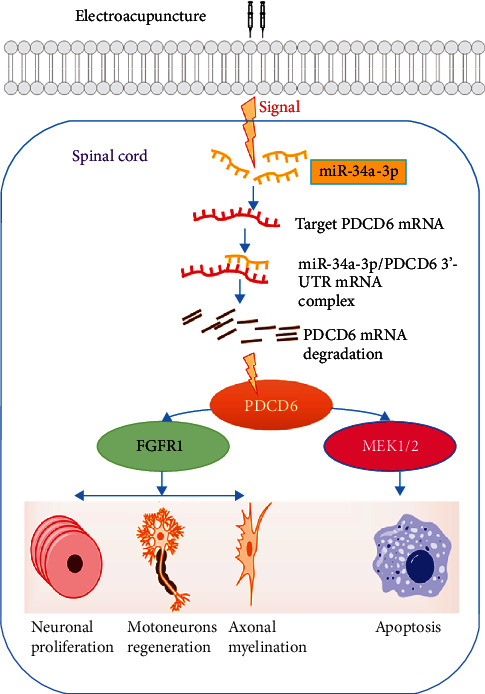
Mechanism of the effect of EA on the miR-34a-3p/PDCD6 axis in SCI.

Axonal myelination is pivotal for the normal functioning of the central nerve system [47]. To comprehensively evaluate the potential of miR-34a-3p as a remyelinative agent, we measured the post-SCI expression of MBP, an established marker for myelination [48], as well as other two important markers NF-200 and GFAP, at protein level. As shown in Figures 6(a)–6(c), the post-SCI expression of MBP and NF-200 was increased in response to EA or miR-34a-3p treatments while the reduced GFAP expression was observed after EA or miR-34a-3p treatments (Figures 6(a) and 6(d)). Immunofluorescence assay results were almost consistent with the WB analyses and indicated that both EA and miR-34a-3p treatments significantly increased the number of axons with positive NF-200 staining and reduced that with positive GFAP staining when compared with the SCI group. These observations (Figures 6(a)–6(g)) suggested that miR-34a-3p may facilitate post-SCI axonal myelination through promoting the expression of NF-200 and MBP and suppressing GFAP. Finally, we visualized post-SCI remyelination in response to miR-34a-3p through TBS. As shown in Figure 6(h), an obvious loss of myelination was observed in both SCI and SCI+NC-agomir groups when comparing the uninjured controls. However, the myelinated cells were regained after miR-34a-3p or EA treatments, either individually or in combination (Figure 6(h)). Consistent with the above data, miR-34a-3p exhibited a promyelinating role which was similar to the effect of EA stimulation (Figure 6(i)). The effect of miR-34a-3p and EA combination treatments were stronger than individual treatments (Figure 6(i)). As shown in the electron microscope images (Figure 6(j)), myelin compaction was observed in post-SCI rats that received either EA or miR-34a-3p treatments; in contrast, fragmented myelin was found in post-SCI rats without the preceding treatments (Figure 6(j)). Our current study described a potential miR-34a-3p/PDCD6 axis that promotes the post-SCI recovery by reducing apoptosis (through MEK1/2) and promoting various neuron functions such as neuronal proliferation, motoneurons regeneration, and axonal myelination (through FGFR1) (Figure 7).

## 4. Discussion

The homeostasis of numerous miRNAs is perturbed after the induction of SCI. Depending on their downstream targets, dysregulation of these epigenetic regulators might result in either alleviated or aggravated post-SCI condition [49]. In the current research, we first found that miR-34a-3p might target PDCD6 and miR-34a-3p-regulated NSCs differentiation *via* suppressing PDCD6. Considering the proapoptotic nature of PDCD6 [18], we hypothesized that miR-34a-3p might exert an opposite effect by negatively regulating PDCD6, in consistency with previous reports [30, 31]. Our subsequent dual-luciferase reporter assay and WB analysis confirmed our conjecture. Based on these evidences, we further proposed that miR-34a-3p could be beneficial to post-SCI regeneration in the SD rat model.

EA has been proven to facilitate post-SCI neurologic and functional recoveries, but the mechanism remains to be clarified. In this study, we evaluated the involvement of the miR-34a-3/PDCD6 regulatory axis in the therapeutic effect of EA treatment in an animal SCI model. The results revealed that miR-34a-3p promoted NSC proliferation and neuronal differentiation and regulated SCI-associated signaling pathways by targeting the PDCD6 axis. Interestingly, EA regulation of the miR-34a-3p/PDCD6 axis facilitated post-SCI neural regeneration and axonal myelination in rats. All these suggest that miR-34a-3p plays a critical part in EA therapy for SCI and that miR-34a-3p may be an attractive therapeutic target for SCI treatment.

To further clarify the involvement of neuron-specific markers in the miR-34a-3p/PDCD6 regulating network, we evaluated the expression of FGFR1, MEK1/2, GFAP, MBP, and *β*III-tubulin in response to forced (transient) miR-34a-3p overexpression and found that aside from suppressing PDCD6 expression, miR-34a-3p promoted FGFR1, *β*III tubulin, and MBP protein levels, while suppressing the protein expression of GFAP and activation of MEK1/2. Aside from its important role in SCI [46], activation of MEK1/2 has long been considered accountable for apoptosis in SCI, because ERK1/2, an important upstream regulator of various apoptotic factors (including caspase 9 and BCL2-family protein Bim), was directly activated by MEK1/2 [50]. The abovementioned evidences suggested that in our current study, the promoted post-SCI regeneration and neural proliferation might be attributed to the miR-34a-3p-induced inhibition of the MEK1/2 signaling. In contrast, delivery of FGFR1 ligand to the lesion site of the SCI was shown be beneficial to post-SCI recovery and neural proliferation [45]. Importantly, FGFR1 is essential for neural proliferation and was upregulated in differentiating neural cells [51]. These observations agreed with our current study showing that miR-34a-3p-induced FGFR1 elevation was accompanied by improved neuronal proliferation, motoneurons regeneration, and axonal myelination.

Currently, there are no solid evidence for the interaction between PDCD6/miR-34a-3p and the preceding neuron markers. However, in cancer cell lines, PDCD6 could mediate the inhibition of ASK1/JNK pathway through Raf-1 (Raf-1 protooncogene, serine/threonine kinase), thereby increasing tumor sensitivity to chemotherapy, and Raf-1 was required for MEK/2 activation under proapoptotic conditions [52]. Therefore, Raf-1 might serve as a bridge that connect PDCD6 and the activation of MEK1/2, whereby they work in concert to regulate apoptosis.

The therapeutic efficacy of EA has been validated in various SCI rat model, including promoting motor function [53], improving neuronal function [54], and facilitating the proliferation and differentiation of oligodendrocyte precursor cells [55]. However, the mechanism underlying the therapeutic effect EA is not well elucidated. A previous study investigated the expression profiles of miRNAs involved in EA-treated SCI rats and demonstrated the potential mechanism and functional role as well as the potential as therapeutic targets of miRNAs in SCI rats [56]. Studies have also indicated that EA downregulates the expression of miR-449a to increase NSCs proliferation and promote neuron survival [17]. The miR-214 was also found to be a target of EA in SCI [33]. Herein, we evaluated whether the therapeutic potential of EA could be driven through the miR-34a-3p/PDCD6 axis in post-SCI recovery. We demonstrated that EA as well as the miR-34a-3p/PDCD6 axis facilitates post-SCI neural regeneration and axonal myelination in rats and these effects were further promoted by the combined treatment with EA and miR-34a-3p-agomir, indicating that the miR-34a-3p/PDCD6 axis mediates the therapeutic effect of EA. This study is the first of its kind to establish the therapeutic regulatory role of EA on miR-34a-3p in post-SCI recovery.

In summary, we demonstrated that this pathway is a target for EA. These results might provide a novel perspective for developing therapeutic strategies against SCI and shed light on the pathogenesis and contribute in the in-depth researches on post-SCI recovery. However, this study still has room for improvement. miRNAs may present differential expression in different cell types, which can directly or indirectly regulate target mRNAs. In addition, a miRNA may have multiple target mRNAs, resulting in the difficulty in evaluating the interaction between its target mRNAs. In addition, accurate acupoint positioning is also crucial for EA. Therefore, further studies with a larger sample size are needed to clarify the relationship between EA, miRNA changes, protein expression, and spinal cord injury recovery.

## Data Availability

All relevant data are within the paper.
